# Water

**Published:** 1884-07

**Authors:** 


### WATER.

Water is the great mechanical power in nature. It is the great leveler, it moves mountains and fills valleys. All our stratified rocks, sandstones, slate and limestones were formed by the action of water. To the solvent power of water and its chemical action we owe our useful minerals, our metallic deposits, our iron, copper, zinc, gold and silver ores, and even coal. To its physical properties--its relation to heat and cold--we owe all phenomena of clouds, dew, rain, fog, snow and frost. It supports the plants, brings them their mineral food from the soil and protects them from excessive heat. Animals are equally dependent upon it. Yet, after all, it is only the sun ; it is sun-power that moves everything in the world, and water is merely the sun's agent. The loss of water would produce the same condition of things on earth as we notice now in the moon. Although we have such a vast quantity of water, yet it is only one two hundred and forty-thousandth part of the the errth, or 0.0242 per cent. The crystalline rocks in the earth's surface now contain a larger quantity of water than this, and the moment our earth cools enough to absorb four-thousandths of one per cent. of moisture, the ocean will disappear. If we should lose our atmosphere also, the pores in the rocks will receive it by gravitation.

A French anatomist, Mons. Luys, has found that when a person lies down or stands upon his head the brain changes its position in the skull in obedience to the law of gravitation. The movements take place slowly, five or six minutes being required for the brain to adapt itself to a new attitude of the body.

